# Deep learning-based physiological risk stratification in night-shift hospital workers

**DOI:** 10.1038/s41598-026-43982-y

**Published:** 2026-03-16

**Authors:** InHo Lee, SangHee Hong, JuneHee Lee, HwaYoung Lee, SoonChan Kwon, YoungSun Min, EunChul Jang, JeongBeom Lee

**Affiliations:** 1 Department of Occupational and Environmental Medicine, Soonchunhyang University Cheonan Hospital, Cheonan, Republic of Korea; 2 Department of Physiology, College of Medicine, Soonchunhyang University, Cheonan, 31151 Republic of Korea; 3 Department of Medical Sciences, Graduate School, Soonchunhyang University, Asan, Republic of Korea; 4 Department of Occupational and Environmental Medicine, Soonchunhyang University Hospital, Seoul, Republic of Korea; 5 Department of Psychiatry, Soonchunhyang University Cheonan Hospital, Cheonan, 31151 Republic of Korea; 6 Advanced Pharmacology and Physiology Integrated Sciences (AXIS), College of Medicine, Soonchunhyang University, Cheonan, 31151 Republic of Korea

**Keywords:** Night-shift, PHATE-NAM analysis, Accumulated night exposure, Circadian misalignment, Engineering, Health care, Health occupations, Medical research

## Abstract

This cross-sectional study of 2,250 hospital workers attempted to identify potential physiological risk structures for night workers by utilizing the nonlinear manifold learning framework. Unlike conventional mean-centered linear analysis, this study applied the Potential of Heat-diffusion for Affinity-based Transition Embedding (PHATE) for topological visualization and Neural Additive Models (NAM) to predict interpretable risk. The ‘cholesterol homeostasis profile’ was defined as the main latent indicator. Analysis showed that the systemic metabolic load (PHATE-1) was higher after age correction, despite the night-shift group being younger than the non-night shift group (p < 0.001). This axis showed a positive correlation with body mass index (r = 0.71) and triglyceride (r = 0.56). The NAM model captured the risk slope with an average AUC of 0.864 (range 0.832–0.901) and confirmed a nonlinear risk transition when it exceeded the triglyceride 2 mmol/L threshold. The predicted risk of night workers in the highest risk cluster (cluster 1) was 0.71, and the metabolic load and cholesterol homeostasis profile risk were increased. Ultimately, the PHATE-NAM framework moves beyond traditional group averages. It provides a purely data-driven way to pinpoint individual physiological vulnerabilities, giving hospitals the exact evidence needed to design safer, more sustainable shift-work environments.

**Supplementary Information:**

The online version contains supplementary material available at 10.1038/s41598-026-43982-y.

## Introduction

Continuous care through 24-hour night shift work, maintaining a care system, requires a harsh biological price for hospital workers^1,2^. Night shift disrupts the natural connection between a person’s biological clock causing sleep deprivation^3^. Night shift work is known to be associated with adverse metabolic and cardiovascular health effects^4,5^. The irregular blood pressure and metabolic changes observed in night shift workers indicate that the body is undergoing long-term physical load^3,5^. This evidence is also shown in prominent international research reports. Major international organizations, including the World Health Organization (WHO)^6^, the International Labor Organization (ILO)^7^, and the National Institute for Occupational Safety and Health (NIOSH)^8^ have characterized night shift work as an occupational hazard. They point out the need for precise and individualized tracking systems to identify and monitor the physiological deterioration caused by night shifts.

Up to now, previous studies have focused on identifying differences in group averages between night and non-night workers^9^. Although these analytical methodologies have contributed to understanding the overall risk level of night work, it has been difficult to fully explain how consecutive working days or complex job factors are associated with the physiological variability of individual workers^10–12^. In addition, previous studies focused only on numerical changes in specific indicators due to night work, and there were limits to analyzing the heterogeneity of high-dimensional physiological responses in which biological indicators are intricately intertwined^13^. Night work affects metabolism, hemodynamics, and periodic biomarkers, and their complex and nonlinear interactions adversely affect workers’ health. Considering the characteristics of a cross-sectional study^14^, instead of analyzing the causal outcomes of certain diseases driven by night work^2,15,16^, this study aims to explore what correlation patterns and interactions the biochemical indicators of workers form within the health risk landscape associated with potential night work. This perspective is essential for capturing individual vulnerabilities that have been eclipsed by group averages and for identifying the diversity of physiological responses within the same working environment^5^.

**Fig. 1:**
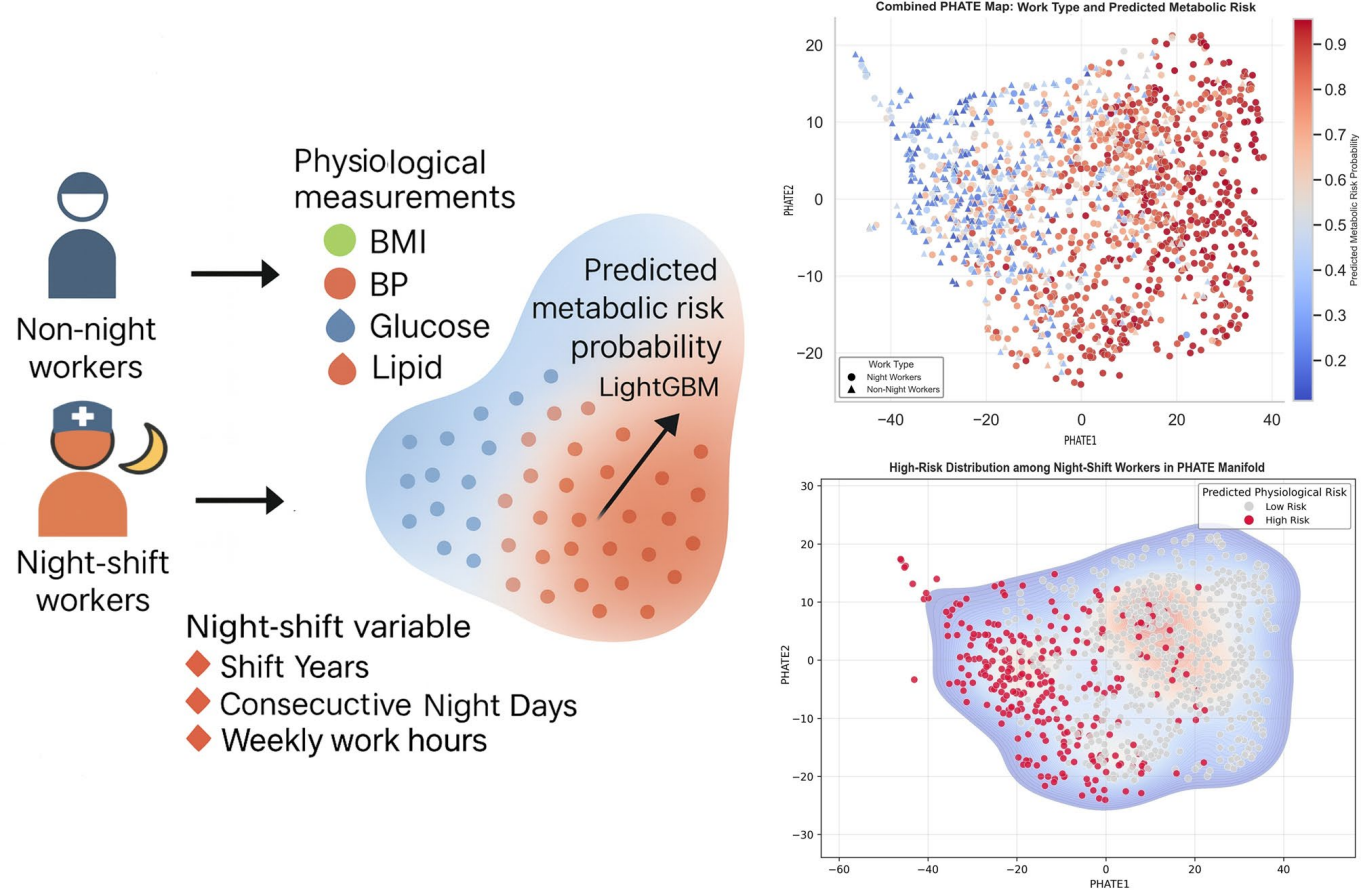
Conceptual overview of the PHATE–NAM analytical workflow. This scheme illustrates the analysis process that constitutes a potential risk landscape. The probability of metabolic risk was estimated based on LightGBM using physiological variables such as BMI, blood pressure, and geological indicators, as well as occupational variables related to night work. The right panel displays the results of projecting these multidimensional profiles into a low-dimensional space using the PHATE technique. In the formed latent space, PHATE-1 reflects the systemic metabolic burden, and PHATE-2 reflects the cholesterol homeostasis pattern. Each point represents the physiological position of an individual in that space.

To systematically analyze the nonlinearity and complexity of the patterns of indicators and physiological data, we utilized an analysis framework (Fig. 1) that combines the Potential of Heat-diffusion for Affinity-based Trajectory Embedding (PHATE) technique and Neural Additive Models (NAM)^17,18^. PHATE shows workers’ ‘physiological maps’ to compress diversified physiological data into a visualizable environment. This enables the visualization of natural interactions between physiological indicators and transfer patterns for the risk of working at night^17^. NAM is an ‘interpretable glass box’ model, and unlike ‘black box’ AI, which has an opaque decision-making process, it can identify risk probabilities nonlinearly. In fact, it is essential to secure the reliability and validity of the AI model in the decision-making stage for application in a clinical setting, so the nonlinear predictive performance of deep learning and the interpretability of the linear model can be secured at the same time^18,19^. This step-by-step analysis is significant in that it can help managers make data-based decisions in judging the risk of working at night in the actual clinical field, beyond simply enhancing predictive performance^20–22^.

The major latent axis derived from PHATE analysis, the Cholesterol Homeostasis Profile, is based on the evidence that the stable regulatory ability of lipid metabolic indicators such as triglyceride, LDL, and HDL cholesterol correlates with night work^23,24^. This reflects the clinical significance of triglyceride to HDL ratio, a well-known indicator of metabolic homeostasis and insulin resistance in our preceding study^24^. Therefore, instead of looking at lipids as isolated levels, we identified them as indicators of the body’s inherent ability to buffer PHATE-derived metabolic stress.

This study aims to verify that night workers form a unique potential physiological risk structure that distinguishes them from non-night workers through nonlinear representation learning. We hypothesized that night shift exposure is associated with the formation of certain high-risk clusters within the latent space beyond mere deterioration of indicator values, and that metabolic risk including cholesterol homeostasis profiles is a major factor characterizing risk structures. The results of this study provide an academic basis for preemptively identifying high-risk groups within the organization and establishing a sustainable working environment design and precise preventive health management system that takes into account the physiological limitations of night shift workers within the hospital’s 24-hour operating system.

## Methods

### Study participant dataset

A total of 2,250 hospital employees were included in this study, comprising 1,151 night-shift workers and 1,099 non-night-shift workers who participated in the Soonchunhyang University Hospital Occupational Health Program (2025). Data were obtained from the Health Screening Program and integrated with the institution’s Occupational Health Dataset, comprising real-world electronic medical records (EMR). Baseline demographic and occupational characteristics of night-shift and non–night-shift workers are summarized in Table 1.

**Table 1:** Baseline characteristics of night-shift and non–night workers.

Variable	Night-shift (*n* = 1,151)	Non–night (*n* = 1,099)	*P*-value
Age (years)	30.9 ± 8.8	40.7 ± 11.0	< 0.001
BMI (kg/m²)	22.4 ± 3.5	24.5 ± 4.0	< 0.001
Waist circumference (cm)	72.1 ± 9.6	84.2 ± 8.2	< 0.001
SBP (mmHg)	116.1 ± 12.6	121.4 ± 12.4	< 0.001
DBP (mmHg)	72.7 ± 9.7	76.6 ± 9.3	< 0.001
Fasting glucose (mg/dL)	94.8 ± 10.0	100.3 ± 19.8	< 0.001
Triglyceride (mg/dL)	88.3 ± 58.8	129.7 ± 104.7	< 0.001
HDL-cholesterol (mg/dL)	66.8 ± 14.9	59.4 ± 16.3	< 0.001
LDL-cholesterol (mg/dL)	113.1 ± 29.2	115.9 ± 34.8	0.084
Job role, n (%)			
Nursing staff	483 (42.0)	444 (40.4)	**—**
Clinical staff	299 (26.0)	293 (26.7)	**—**
Administrative staff	219 (19.0)	215 (19.6)	**—**
Facility / support staff	150 (13.0)	147 (13.3)	**—**

Values are presented as mean ± standard deviation or number (%). SBP, systolic blood pressure; DBP, diastolic blood pressure; BMI, body mass index.

To minimize selection bias and ensure the ecological validity of the study, we adopted a total census approach, enrolling the entire cohort of eligible employees who participated in the Soonchunhyang University Occupational Health Program (2025). Although the sample size was determined by this comprehensive enrollment strategy, a post-hoc power analysis confirmed the statistical robustness of the design. The final sample size (*n* = 2,250) achieved a statistical power of > 99.7% (1-β > 0.99) to detect even small effect sizes (Cohen’s d = 0.2) at a significance level of α = 0.05, ensuring sufficient sensitivity to identify physiological differences between groups.

The inclusion criteria were as follows: (1) full-time employment for more than one year, (2) completion of fasting biochemical examinations, and (3) availability of verified shift-work records.

The exclusion criteria were as follows: (1) missing key biochemical indicators and (2) current active treatment for chronic metabolic or endocrine diseases.

### Ethical approval and data collection

All participants provided written informed consent, and the study protocol was reviewed and approved by the Soonchunhyang University Cheonan Hospital Institutional Review Board (IRB No. SCHCA-2025-10-010). All procedures involving human participants were conducted in accordance with the relevant guidelines and regulations, including institutional policies and the principles of the Declaration of Helsinki. The dataset included routine biochemical and hemodynamic parameters such as triglyceride (TG), total cholesterol (TC), fasting glucose, aspartate aminotransferase (AST), alanine aminotransferase (ALT), systolic blood pressure (SBP), and diastolic blood pressure (DBP). All laboratory tests were performed under fasting conditions, following standard hospital protocols.

## Data preprocessing and statistical analysis schemes

Cleaning and standardization were performed for data analysis. Continuous variables were normalized using Z-score transformation, and outliers were processed according to the interquartile range (IQR) standard (1.5 × IQR). As a result of checking the entire data, missing values accounted for less than 1.5%, for which a simple distribution-based imputation method was applied. In order to prevent overfitting of the model and increase the generalization performance, five-fold cross-validation (25 repetitions) was applied, and stability was verified based on the average and standard deviation of the calculated performance indicators.

For analysis, clusters were first identified based on the latent distribution inherent in the data using VaDE, and then PHATE was applied to project the data for those clusters into the latent space. Using this integrated technique, we were able to visualize the high-risk transition process.

All data preprocessing and integration processes were performed in the Python 3.11 environment using general-purpose analysis packages, including pandas, NumPy, and scikit-learn. The original dataset is restricted from external disclosure according to the agency’s information protection policy, but it is accessible through the corresponding author (Lee Jeong-beom, leejb@sch.ac.kr) for reasonable research purposes.

## Method details

### Nonlinear manifold embedding (PHATE)

All continuous physiological variables underwent Z-score normalization prior to analysis to ensure scale consistency. To explore the potential structure formed by several physiological markers, we applied the PHATE algorithm (Python package phate, v3.0) to a standardized data matrix. PHATE constructs adaptive diffusion graphs based on local data density, allowing us to visualize continuous physiological gradients across the data instead of discrete classification. Similarities among participants were calculated through adaptive Gaussian kernels and converted to transition probabilities, while potential distances were calculated by applying the diffusion time (t) parameter (see Supplementary Methods: Mathematical Formulation of PHATE). To ensure the structural performance of the embeddings, a sensitivity analysis to sweep was performed by setting diffusion times (t) to 10, 30, 50, and attenuation to 20, 40, and 80 (Supplementary Table S1). To clarify the biological importance of the derived latent axes, quantitative correlation mapping was performed, and the final model parameters were selected based on the stability of the latent structure (see Supplementary Methods: Mathematical Formulation of PHATE).

Furthermore, a potential gradient distribution analysis was performed to quantify the slope of risk transfer in PHATE, and local gradient refers to the speed of physiological change and the sensitivity of specific conditions under night work and homeostasis. By associating this with occupational characteristics such as cumulative night work period and intensity of labor, we analyzed how a specific work type promotes the deterioration of health indicators.

## Risk probability modeling (LightGBM)

In order to quantitatively confirm how physiological data interact with job factors in the latent space, risk probability was modeled using LightGBM (see Supplementary Methods: LightGBM for the model formulation). A high-performance risk estimation was performed to build a continuous risk landscape and to define the risk density across the manifold. In addition, in order to prevent errors in circular reasoning, a practical interpretation of the physiological mechanism was confirmed through transparent Neural Additive Models (NAM). To prevent overfitting of the model and to ensure generalization performance, strict iterative stratified 5-fold cross-validation (a total of 25 runs) was applied instead of the traditional single-out. The performance evaluation of the model used the receiver manipulation characteristic curve area (AUC) as the main indicator, and the predictive stability of the model was secured through cross-validation. Through this, the calculated average predictive probability was projected onto the PHATE embedding to build a continuous risk landscape, which was used to visualize the metabolic risk changing along the PHATE-1 axis.

### Deep interpretable risk learning (NAM)

To transparently interpret nonlinear relationships between variables beyond the limits of tree-based models, a PyTorch-based (v2.3.1) NAM was implemented. Considering that the night shift group, which is a significant demographic difference, is younger than the day work group, we explicitly included age and sex as input covariates. In addition, we utilized NAM to mathematically separate these effects. Since NAM calculates the contribution by each feature through an independent function, metabolic risk associated with biochemical indicators can be assessed together with metabolic indicators, work intensity index, and PHATE coordinates regardless of aging effect. Each physiological and job variable is modeled through an independent subnetwork and then additionally combined to produce a final output (see Supplementary Methods: Structure of Nonlinear Additive Model (NAM)). As input variables, metabolic indicators, work intensity index, and PHATE coordinates were included. For reproducibility, all deep learning models were trained with a fixed random seed (seed = 42).

For our high-risk cutoff, we isolated the top 30% of the probability distribution. We chose this specific metric as a slightly tighter variation of the classic top-tertile split (roughly 33%) widely used across epidemiological studies^25^. Furthermore, we confirmed through sensitivity checks that the core topological features and cluster compositions remained stable around nearby thresholds, demonstrating that our results are robust to minor variations in the cutoff definition. Zeroing in on this upper tail is simply a more efficient way to target clinical interventions^26^. We also had to ensure model stability. We ran repeated cross-validation for this, replicating our LightGBM protocol. This step guarantees that the variable contributions we calculate are universal, rather than just noise from a single biased sample. To justify the nonlinear method, we put NAM up against standard Logistic Regression and Random Forest baselines.

NAM enables visual tracking of nonlinear effects while maintaining predictive performance. When comparing model performance, the results support that NAM is optimized in that it provides a high level of accuracy and interpretability required in the clinical field (Supplementary Table S2). That is, NAM guarantees a level of accuracy similar to that of existing black box models and provides intuitive identification of nonlinear patterns in which each variable contributes to the risk probability.

## Latent cluster identification (VaDE)

After topological embedding, we applied a variational deep embedding (VaDE) framework combining a variational autoencoder (VAE) and a Gaussian mixture model (GMM) (see Supplementary Methods: Loss Function for Variational Deep Embedding (VaDE). Using PHATE-induced latent coordinates input from the encoder, we compressed the data into a low-dimensional space and estimated stochastic cluster membership via GMM. Specifically, to verify the statistical stability of the identified cluster structure, iterative evaluations were performed using different initialization seeds, and the reproducibility of subtype induction was verified using the Adjusted Rand Index (ARI). For the final reported results, the model was initialized with a fixed random seed (seed = 42) to ensure strict reproducibility. Detailed hyperparameters and architectural configurations for all implemented models (PHATE, LightGBM, NAM, and VaDE) are summarized in Supplementary Table S3. All deep learning analyses were performed using PyTorch (v2.3.1), while visualization and clustering were performed using Scikit-learn (v1.5.0). The PHATE-NAM-VaDE code used in this study can be provided by the corresponding author upon reasonable request, and all key algorithms were implemented using the aforementioned open-source packages (PHATE, LightGBM, and PyTorch). Researchers wishing to access the data must go through a formal review process of their research plan to comply with privacy regulations before requesting an anonymized dataset.

## Results

### Global PHATE topology of physiological variation in hospital staff

**Fig. 2:**
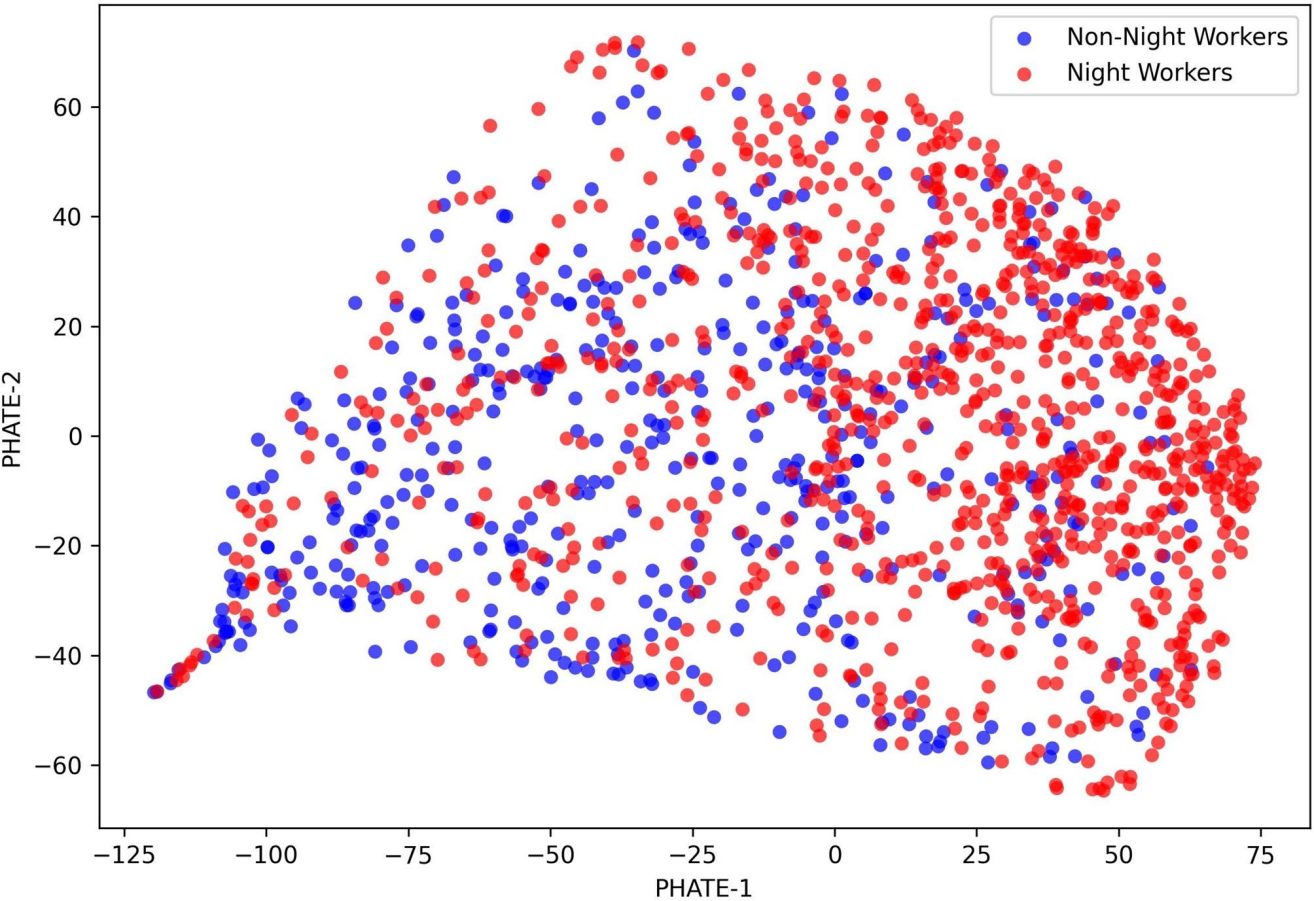
Physiological distribution of hospital workers mapped onto the PHATE manifold. This figure is a visualization of the physiological distribution of hospital workers. Night workers (red) and day workers (blue) are distinct from each other in the phase space. The PHATE-1 axis reflects the systemic metabolic burden and shows positive correlations with body mass index (r = 0.71), systolic blood pressure (r = 0.67), and triglycerides (r = 0.56). The PHATE-2 axis represents the cholesterol homeostasis profile, which is defined as the conflicting contributions between LDL (r = 0.67) and HDL (r = 0.51). The density contour was calculated by Gaussian kernel density estimation. It means that higher densities indicate higher phase concentrations of individuals in the region. The night shift distribution was significantly to the right in the PHATE-1 axis direction. This means that structural changes associated with increased metabolic burden have appeared (p < 0.001).

The PHATE-1 axis, representing ‘Metabolic Load’, showed positive correlations with body mass index (BMI, *r* = 0.71), systolic blood pressure (SBP, *r* = 0.67), and triglycerides (TG, *r* = 0.56). The PHATE-2 axis, defined as the cholesterol homeostasis profile, showed significant correlations with LDL cholesterol (*r* = 0.67) and HDL cholesterol (*r* = 0.51).

The Gaussian kernel density estimation (KDE) contours in Fig. 2 show a clear contrast. The night shift group had a significantly higher distribution on the PHATE-1 axis compared to the non-night shift group (*p* < 0.001). This reveals a co-elevation pattern where metabolic burden markers, such as triglycerides, blood sugar, and liver enzymes, rise together in the night shift cohort. However, the wide distribution of PHATE-2 levels confirmed that significant physiological heterogeneity, driven by individual cholesterol homeostasis control, exists even within the night shift group itself.

### Spatial distribution of predicted metabolic risk

**Fig. 3:**
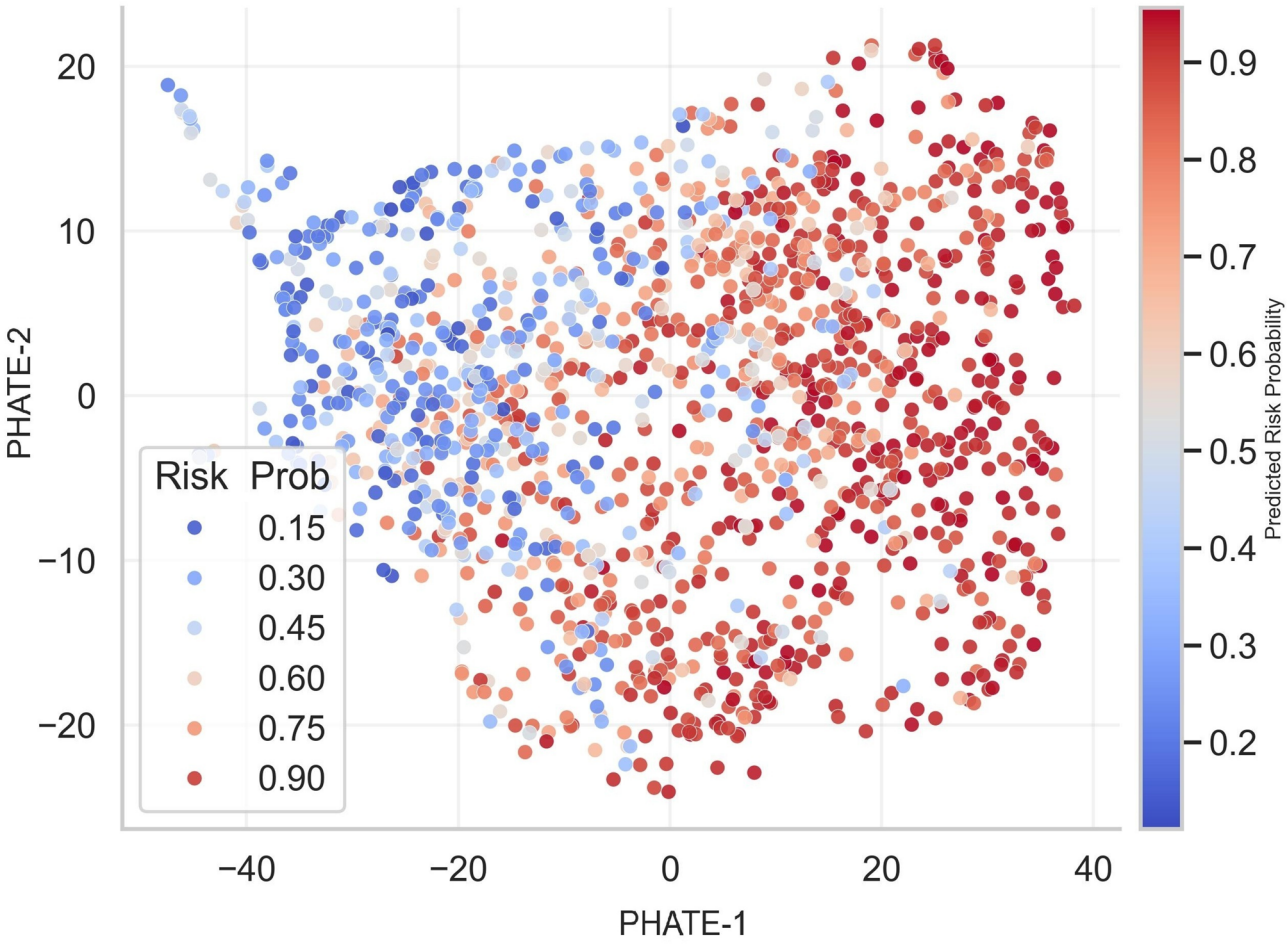
Projection of predicted metabolic risk across the global PHATE landscape. This figure shows the result of projecting the predicted risk probability into the PHATE latent space using a lightweight gradient boosting machine (LightGBM) model. The axis consists of a dimensionless latent distance that preserves the global structure of the physiological state, PHATE-1 reflects systemic metabolic load, and PHATE-2 reflects cholesterol homeostasis-related variations. Regions with a predicted probability of 0.70 or more (red series) tend to be concentrated on the right side of the manifold, that is, in the section with high metabolic load. The average AUC of the model was 0.886 (standard deviation = 0.018), confirming that the risk distribution in the latent space was stably classified. This nonlinear risk distribution visually presents the change in risk level according to the physiological position in the PHATE space.

As a result of projecting the LightGBM model into the PHATE space (Fig. 3), it was confirmed that a high-risk region was formed in the area with a high PHATE-1 value. The average AUC calculated through repeated layered 5-layer cross-validation was 0.886 (SD = 0.018, range 0.852–0.915), demonstrating that the model stably captures the risk gradient in the latent space. Furthermore, we supported the predictability of the model by analyzing calibration curves that showed a high level of alignment between the predictive probability and the observed risk frequency. As we approach the 45-degree mark, we see that the model provides a reliable risk estimate. In particular, the high-risk group with a predictive risk of 0.7 or more was spatially concentrated and distributed in the right area of the manifold where the metabolic load was concentrated.

### Local occupational topology of night-shift workers

**Fig. 4:**
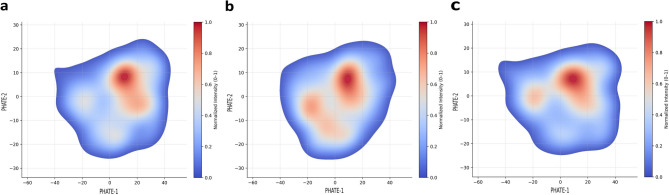
Occupational gradient patterns among night workers in latent space. As a result of the latent slope distribution analysis targeting only night workers, the high-density areas of (**a**) continuous night shift days, (**b**) cumulative working hours, and (**c**) weekly working hours significantly overlapped with the high-risk section of PHATE-1. This pattern was reflected as a nonlinear effect in the NAM model, and the risk probability tended to increase if it deviated from the section.

As a result of the latent gradient mapping analysis (Fig. 4) targeting only night workers, it was found that the high-density areas of (a) consecutive night workdays, (b) cumulative number of working hours, and (c) weekly work hours significantly overlapped with the high-risk areas of PHATE-1. These patterns suggest that accumulated night exposure and long working hours are closely related to physiological stress weighting and show that the PHATE-1 axis effectively captures the changes in biomarkers overall in the night work environment.

### NAM-based interpretable modeling: prediction and explanation

**Fig. 5:**
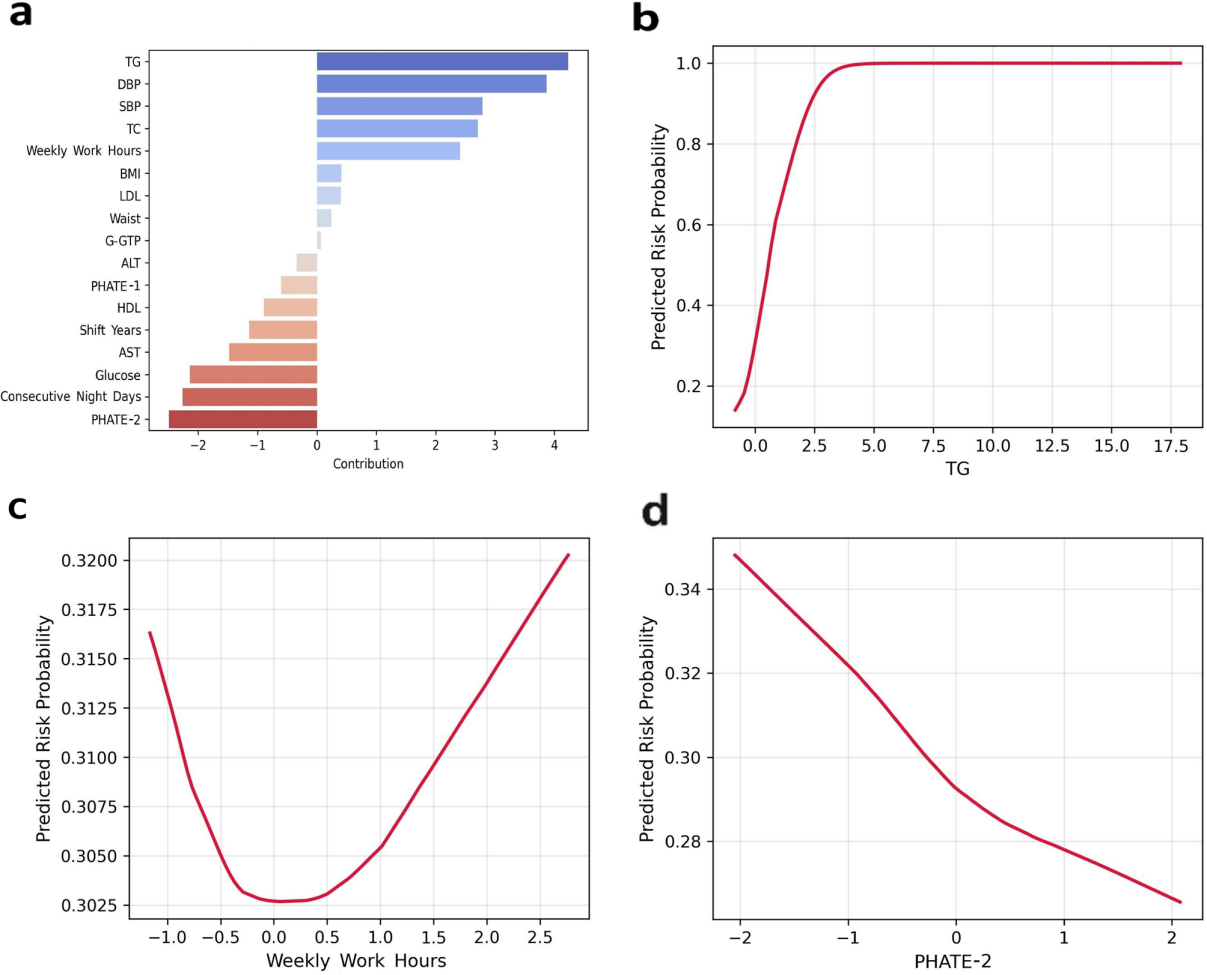
Interpretable risk components derived from the NAM model. (**a**) As a result of analyzing the variable contribution of the Natural Additive Model (NAM), triglyceride (TG), blood pressure (SBP/DBP), total cholesterol (TC), and working hours per week showed a direction associated with increased risk. On the other hand, PHATE-2 (cholesterol homeostasis profile) and continuous working days were associated with a risk reduction trend. (b–d) partial dependence curves representing nonlinear risk changes were presented. Biometric indicators were presented in standard clinical units, and triglycerides were presented in mmol/L, blood pressure was presented in mmHg, and working hours were presented in hours/week. (**b**) A nonlinear increase in risk was observed in the section where triglycerides exceeded about 150 mg/dL (≈ 1.7 mmol/L). (**c**) Working hours per week showed a U-shaped curve with the lowest risk in the 40–50-hour interval. (**d**) As the PHATE-2 value increased, the risk tended to decrease overall.

Variable importance analysis of the NAM model (Fig. 5a) identified triglycerides (TG), systolic and diastolic blood pressure (SBP/DBP), total cholesterol (TC), and working hours per week as major predictors. Compared with the baseline models, the AUC for logistic regression was lower than that of the NAM (Supplementary Table S2). Random Forest showed slightly higher accuracy (AUC = 0.914) than the NAM, but it has the fundamental problem of lacking the interpretability essential for setting clinical thresholds. The NAM is significant in that it achieves a stable mean AUC of 0.864 (SD = 0.018, range 0.832–0.901) while simultaneously ensuring this interpretability.

Partial dependence analysis (Fig. 5b–d) revealed a nonlinear pattern where the predicted risk increased rapidly when TG levels exceeded approximately 2 mmol/L (Fig. 5b) which is ‘borderline high’ category of NCEP ATP III (approx. 177 mg/dL). For weekly working hours, metabolic risk increased as hours deviated from the standardized mean (Z = 0) (Fig. 5c). Furthermore, PHATE-2 levels showed a significant negative linear relationship with predicted risk reflecting the functional efficiency of reverse cholesterol transport (RCT) mediated by the antagonism between LDL and HDL (Fig. 5d).

### Model discrimination and calibration

**Fig. 6:**
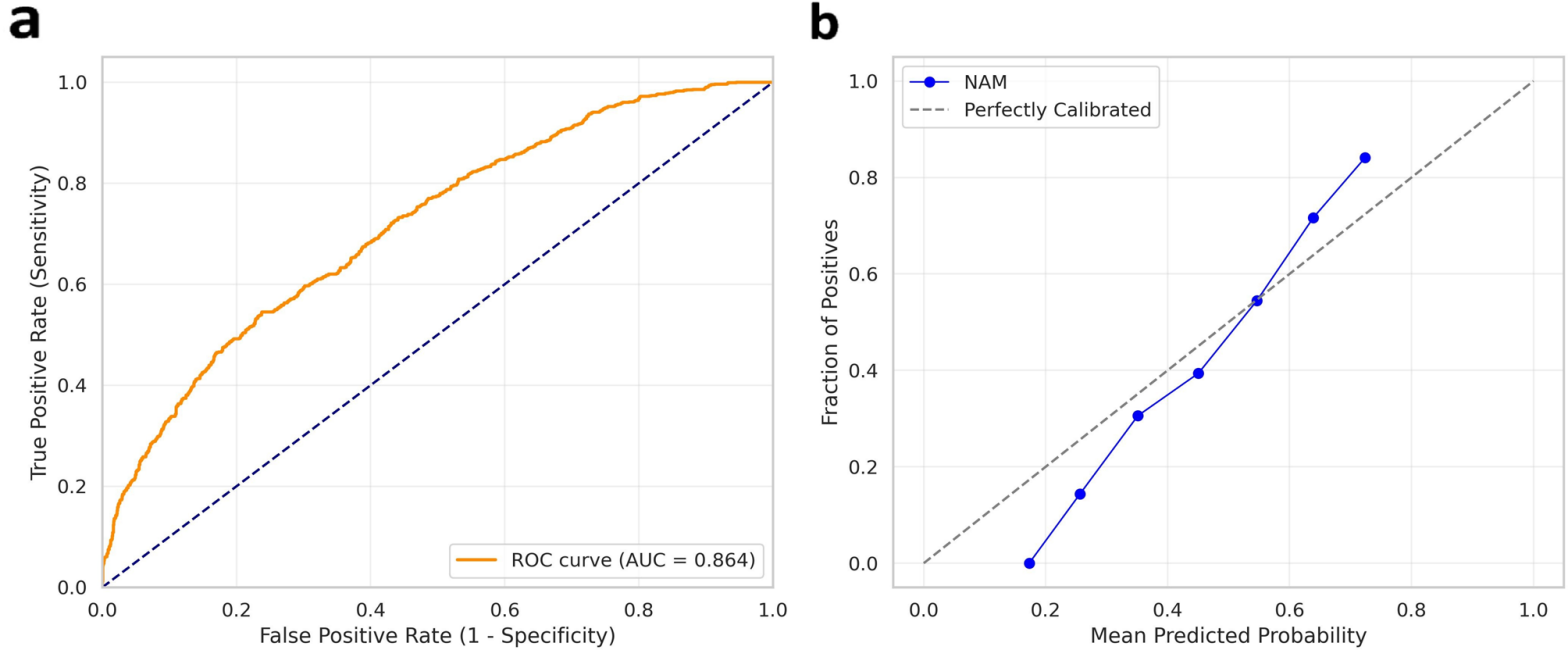
Model discrimination and calibration performance of the NAM framework. (**a**) The ROC curve of the NAM model calculated through repeated cross-validation was presented. The average AUC was 0.864. (**b**) A calibration curve shows the degree of agreement between the predicted probability and the actual observed risk. Overall, good consistency was confirmed between the predicted and observed values.

Average AUC of 0.864 calculated through rigorous iterative cross-validation was recorded (ROC curve, Fig. 6a). The calibration curve (Fig. 6b) shows that the predicted probability closely matches the true event rate, indicating that the model is reliable and not overconfident. The calibration curve (Fig. 6b) shows a high degree of agreement between the risk probability predicted by the model and the actual observed risk distribution.

### High-risk topography within the PHATE manifold

**Fig. 7:**
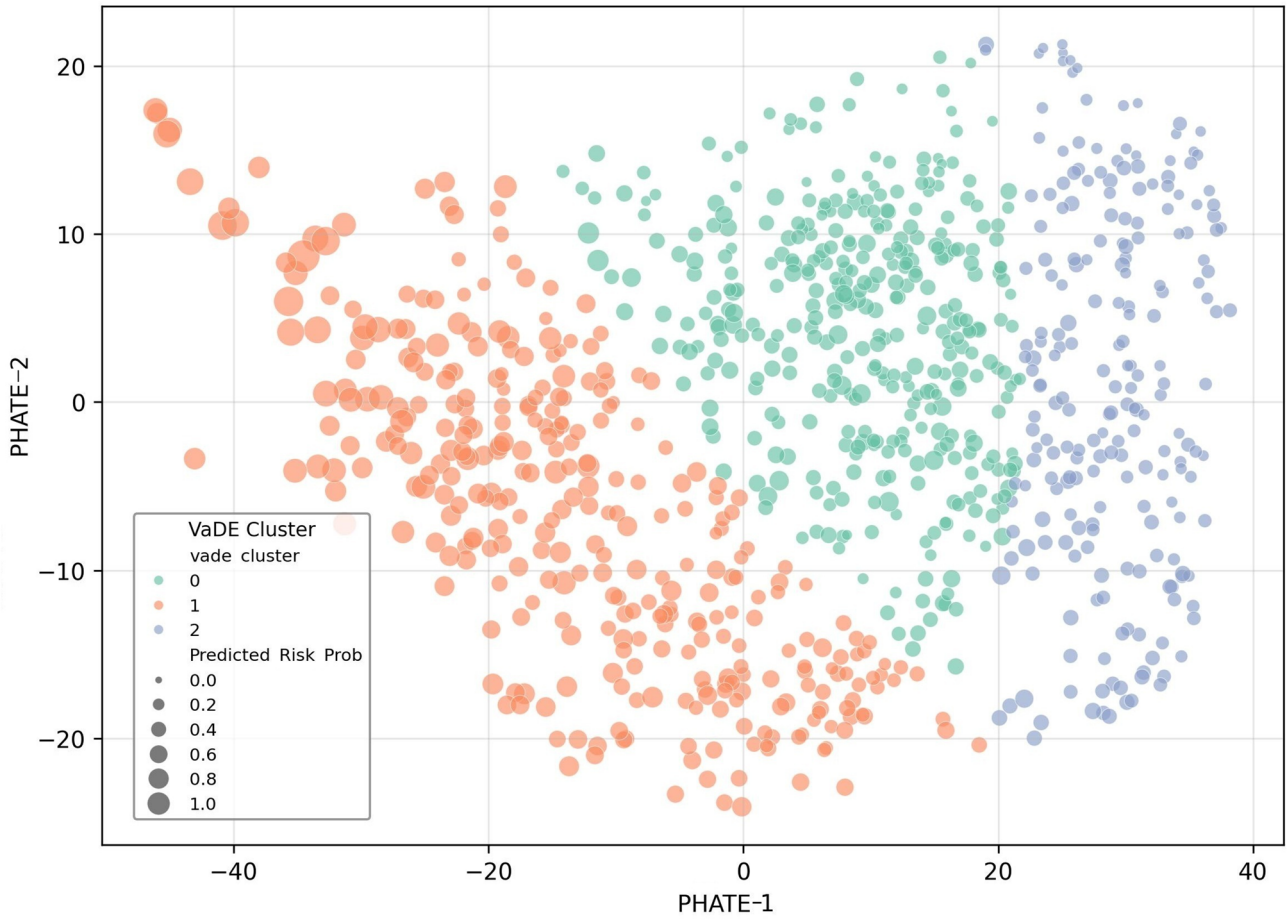
VaDE-based clustering with risk stratification on the PHATE manifold. These are the three potential subtypes of night workers identified by Variational Deep Embedding (VaDE). The axes represent dimensionless potential distances mapping the global manifold geometry, where PHATE-1 captures the gradient of Metabolic Load and PHATE-2 reflects the Cholesterol Homeostasis Profile. Cluster 1 (orange) is the ‘metabolic overload group’ characterized by a negative PHATE-1 gradient, with the highest average predicted risk (0.71). Cluster 2 (green) is a low-risk group with a high PHATE-2 score, which maintains a relatively stable lipid metabolism. The size of the bubble refers to the individual risk probability predicted by NAM (average ARI = 0.858). From a clinical point of view, this stratification enables an occupational health monitoring system based on an individual’s physiological risk level. Cluster 1 is identified as a group with relatively high cardiovascular risk characteristics, regardless of age, and can be a priority for more intensive risk management.

Three major physiological subtypes were identified because of the combination of VaDE clustering and risk probabilities calculated from NAM (Fig. 7). As a result of repeated evaluation with different initialization seeds, the average adjusted Rand Index (ARI) of 0.858 (range 0.689–0.985) was recorded, confirming the high reproducibility and statistical stability of the derived cluster structure.

Cluster 1, which was characterized by a negative PHATE-1 gradient, showed the highest average predicted risk (0.71) among night workers, and concomitant increases in triglyceride, blood pressure, blood sugar and liver enzyme levels were clearly observed. On the other hand, Cluster 2, which was characterized by a high PHATE-2 value, was mainly composed of low-risk groups and showed a pattern of maintaining a relatively stable lipid metabolism despite exposure to night work.

**Fig. 8:**
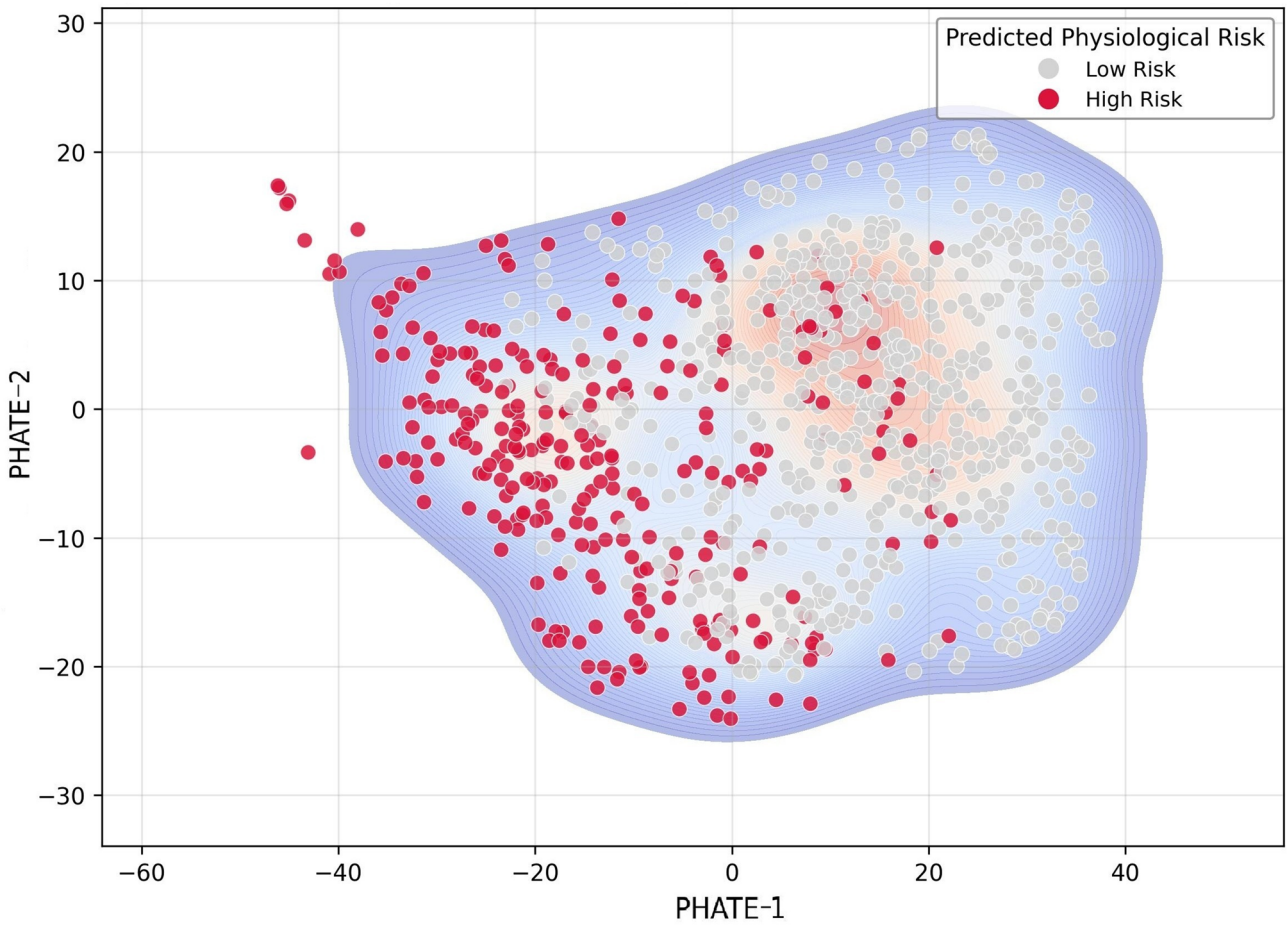
Density contours of high-risk regions within the PHATE space (night-shift subset). The distribution density of high-risk probabilities was visualized through Gaussian kernel density estimation (KDE). Density vertices were formed in the region where PHATE-1 (metabolic load) was negative and PHATE-2 (cholesterol homeostasis profile) was low. The contour lines spatially represent the nonlinear interaction structure between risk factors and present a boundary that can distinguish between sections where the cumulative workload exceeds the physiological buffer range. This distribution pattern suggests the necessity of designing a work structure that considers the transition to the high-density risk section.

Nonlinear interaction structure was identified where risk is most pronounced, specifically manifesting as a high-density convergence in the region with negative PHATE-1 and low PHATE-2 levels (Fig. 8). The most concentrated risk in this context implies a high predicted risk probability. The two axes of PHATE represent that high-risk workers tend to be distributed at the critical point where physiological equilibrium collapses. By analyzing and visualizing the vulnerability of night workers as quantitative patterns within a multidimensional space, this approach explains the physiological mechanism of risk accumulation that is difficult to identify with only a single variable.

## Discussion

This study attempted to confirm distinct physiological adaptations and the pattern of changes in structural patterns under interaction between physiological variables beyond the numerical changes of individual indicators for night work. In Table 1, the night shift group was about 10 years younger than the non-night shift group (30.9 years vs. 40.7 years old), and the superficial metabolic index was also better. This is a typical ‘healthy worker effect’, and the physiological robustness of young workers showed ‘statistical masking’ to cover the cumulative metabolic load according to night shift and working conditions^27,28^. Due to these demographic effects, conventional linear analysis has limitations in detecting the health impact assessment of night shift. In this study, potential data structures were identified through PHATE analysis rather than simple group comparisons to identify structural risks hidden behind the younger age group. Despite the young age of night shift workers, they were distributed along the PHATE-1 axis, which indicates metabolic load, and the exposure to night shift reflected the accumulation of ‘allostatic load’ due to incongruity in biological rhythms, indicating that individuals were located in specific high-risk areas in the potential physiological space^23,24^. The night workers in particular exhibited a wide distribution along the PHATE-2 axis, which, even in the same working environment, could significantly alter the physiological response depending on an individual’s cholesterol homeostasis^24^. Each physiological indicator, blood sugar, blood pressure, and lipid levels, shows the process of systematic integration, indicating topologically that it is a loss of metabolic resilience due to chronic shift work. The spatial slope for the high-risk group identified in LightGBM^29^ indicates that the influence of night shift exposure to key physiological domains is statistically associated^28–30^. This encompasses the multivariate risk correlation structures that univariate analysis often misses^31^. This suggests that beyond traditional single-indicator assessments, a precise industrial health approach that considers topographical flows is needed for various effects, including the working environment of night workers within the latent space.

Regarding the type of work, consecutive night shift days, cumulative working hours, and weekly working hours showed a distinct positive correlation with an increase in metabolic load (PHATE-1)^1,32,33^. This suggests that the physiological position within the potential risk area is not determined simply by the dichotomous exposure to night work, but is complexly related to the intensity of the job and the type of work. Previous studies also showed a significant association between irregular shift work and cumulative exposure and metabolic syndrome indicators, which is in line with the metabolic fluctuation pattern shown in the latent space of this study^2,11,34–36^. The visualization of the latent space goes beyond simply checking the distribution of physiological markers in individuals, and it can be said that it is a tool to show the effect of the system of night work on physiological changes in the human body.

As a result of VaDE analysis, cluster 1, which is a high-risk group, was concentrated in the region where PHATE-1 (metabolic load) was negative and PHATE-2 (Cholesterol Homeostasis Profile) was low (Fig. 7). To ensure the reproducibility and stability of these risk clusters, a VaDE model was constructed using certain latent dimensions and Gaussian mixed model dictionaries. The optimal number of clusters K was determined based on the lower bound of evidence and the silhouette score. This suggests that risk gradients may appear differently depending on individual lipid regulation capabilities even under the same level of metabolic exposure^36,37^. Previous studies have reported that when the regulatory ability of the biological rhythm is deteriorated^38^, it may become more vulnerable to metabolic disease and inflammatory reactions^4,39^, and the instability of the lipid profile observed in this study can be interpreted as a biochemical result reflecting the functional deterioration of these regulatory systems^20,40–42^.

If the global map visualizes the spatial slope of the risk, the NAM is significant in that it identifies the contribution function of each variable and thus increases its applicability in industrial health sites beyond the limitations of the conventional black box model^18,22^. It should be noted that the threshold setting value of TG, 1.7–2.2 mmol/L (150–199 mg/dL), is consistent with the NCEP ATP III guideline, which classifies it as ‘borderline high’^2,43^. This suggests that the NAM model successfully captured the acceleration of the hidden physiological risk before reaching the high-risk criteria due to night work.48 In addition, the U-shaped curve observed in working hours per week showed the lowest risk in the 40-50-hour interval, indicating that out-of-range work patterns are associated with a decrease in physiological stability^1,14,36,37,45,46^.

Beyond the dichotomous analysis of the presence or absence of night work, PHATE shows different physiological responses and structural distributions implemented in the latent space. Although we did not directly measure actigraphy data such as sleep quality or diet in this study, the resilience through cholesterol homeostasis projected on the PHATE-2 axis and the distribution of risk due to night work can be interpreted as the result of the convergence of various factors into physiological indicators^47,48^. Lipid regulatory abnormality acts as a strong biological proxy that integrates the downstream physiological effects of lifestyle factors that have not been measured, serving as a metabolic footprint of biological rhythm disturbance. This is consistent with existing literature suggesting that a decrease in biorhythm stability makes individuals more vulnerable to metabolic disease and inflammatory profile formation^4,39,40^. Therefore, different patterns may appear in this study even for two workers with identical clinical figures for the same metabolic burden. If workers have a strong cholesterol homeostasis profile (high PHATE-2), they may show resilience to metabolic stress. On the other hand, workers lacking this profile who are placed in high-risk belts require preemptive industrial health management even if they work the same night shifts. This suggests that latent coordinates enable preemptive and individualized monitoring of night shift work and metabolic conditions. For night workers, health care requires a precise approach that takes into account inherent physiological capacities, including cholesterol homeostasis profiles, based on an individual’s latent physiological coordinates rather than a uniform standard^9,49,50^.

Therefore, the step-by-step analysis pipeline, from PHATE identifying global topological structures to NAMs calculating the nonlinear contributions of individual variables, is designed to procedurally approach the complexity and interdependence of multidimensional physiological data. This is in line with probabilistic health modeling studies that incorporate predictive risk models and cluster analyses in a cross-sectional study environment^51^, and it can capture frequently overlooked potential risk structures. This analytical approach is useful for identifying the individual vulnerabilities of high-risk workers who are often obscured by group averages^52^, consistent with the findings of large-scale studies of night workers^53^. This provides foundational data for objectively monitoring workers’ physiological loads in environments where night shifts are institutionalized^21,44,54^. While current guidelines from institutions such as EU-OSHA^55^ mainly focus on general shift schedules, the results of this study suggest that individual and detailed approaches tailored to each workplace and worker can be provided to meet global standards.

Although this study provides a data-driven framework for identifying potential physiological risk structures, some limitations should be considered. First, since this is a cross-sectional study of a single university hospital, there are limits to establishing definitive causal relationships. Furthermore, because it is based on a single institution, bias may occur due to the shift work characteristics of a specific hospital or the unique demographic and sociological characteristics of the region. The spatial distribution between the PHATE-NAM risk zones and night shift work provides evidence of spatial associations and physiological gradients rather than temporal causal effects. Health selection effects or issues of temporal precedence cannot be completely excluded. Future longitudinal studies should track individual pattern changes over time on the PHATE manifold, and follow-up verification should be conducted to determine whether the identified high-risk belts and potential slopes accurately predict the actual incidence of metabolic and cardiovascular diseases.

Second, there is a limitation that individual circadian behavior data, such as sleep quality or diet, have not been directly measured. However, the cholesterol Homeostasis Profile defined in this study functions as a physiological endpoint for which these unmeasured various biological background factors finally converge. In particular, the Mean ARI of 0.858 which demonstrates the stability of clustering structures, supports that the identified subtypes reflect a robust physiological structure rather than an accidental distribution of the data.

Third, although there is a possibility of health worker bias (which suggests our findings likely represent a conservative estimate of the true risk), the nonlinear manifold analysis in this study has differentiated value in that it presents specific and immediately viable clinical criteria, such as the triglyceride 2mmol/L threshold, even within the relatively healthy population. These results can be used as practical decision-making support tools for hospital health managers to proactively identify high-risk groups and establish sustainable work designs.

## Conclusion

The analysis system that combines PHATE and NAM clearly demonstrates how the health risks of night workers are organized and spread within the latent space. By identifying the phase structure of the entire data through PHATE and precisely analyzing the contribution of individual variables through NAM, it is possible to identify the number of night shifts, job intensity, and metabolic indicators such as triglycerides and blood sugar that form high-risk areas and connect them. This multidimensional approach deviates from the existing method of relying on the group average and quantitatively demonstrates the degree of risk and why it varies from individual to individual even in the same environment.

The results of this study provide a scientific basis for reducing the physiological load of night workers and designing sustainable work systems. This approach shifts global occupational health management toward data-driven, personalized protection for each worker. It moves beyond uniform corporate and social regulations on night work and provides the scientific evidence needed to update worker health guidelines.

This framework can contribute to establishing practical risk assessment and management strategies in various industrial sites where shift work is essential, including hospitals. Furthermore, precise intervention measures can be tailored by utilizing the latent physiological coordinates of individual workers. Future multicenter cohorts and longitudinal follow-up studies are required to verify the effectiveness of the latent physiological coordinate-based approach proposed in this study.

## Supplementary Information

Below is the link to the electronic supplementary material.

Supplementary Material 1

## Data Availability

The datasets generated and/or analyzed during the current study are not publicly available due to the institution’s Electronic Medical Record (EMR) data protection policy regarding participant privacy. However, the raw data supporting the conclusions of this article are available from the corresponding author (JeongBeom Lee, leejb@sch.ac.kr) upon reasonable request and subject to Institutional Review Board (IRB) approval. To fully comply with the journal’s Code Availability policy and support research reproducibility, the official Python implementation of the analytical pipeline (including PHATE, LightGBM, NAM, and VaDE models) has been made publicly available. The code repository can be accessed at: https://github.com/Crescendo-OEM/DL-Physiological-Risk-Stratification. To ensure permanent accessibility, the version of the code used in this study is also archived at: https://doi.org/10.5281/zenodo.18872957.

## Abbreviations

BMI: body mass index
LDL/HDL: Low-/high-density lipoprotein
G-GTP: Gamma-glutamyl transpeptidase
ALT/AST: Alanine/aspartate aminotransferase
ROC: Receiver operating characteristic
AUC: Area under the curve
