# Supplementary material for: Deep learning-based physiological risk stratification in night-shift hospital workers
Source: Sci Rep. 2026 Mar 16;16:13686. doi: 10.1038/s41598-026-43982-y (PMC13125554; doi:10.1038/s41598-026-43982-y)
Supplement: Supplementary file 1 — Supplementary Material 1 [file 41598_2026_43982_MOESM1_ESM.docx]

**Title: Deep Learning-Based Physiological Risk Stratification in Night-Shift Hospital Workers**

**Authors:** InHo Lee, SangHee Hong, JuneHee Lee, HwaYoung Lee, SoonChan Kwon, YoungSun Min, EunChul Jang, JeongBeom Lee

**Supplementary methods**

**Mathematical Formulation of PHATE**

This section details the mathematical foundation of the Potential of Heat-diffusion Affinity-based Transition Embedding (PHATE) used in this study.

First, pairwise similarities between individuals were computed using an adaptive Gaussian kernel to capture local data structures:

$$K_{ij}=\exp(-\frac{\mid\mid x_{i}-x_{j}\mid\mid^{2}}{\sigma_{i}\sigma_{j}}),$$

*(Eq. S1)*

where $x_{i}$and $x_{j}$ are data points, and $\sigma_{i}{, \sigma}_{j}$ are the adaptive bandwidths (local scaling parameters) for each point.

These affinities were converted to transition probabilities $P=D^{-1}K$. After applying a diffusion process with the time parameter $t$, the potential distance (information distance) between two data points was calculated as:

$$\Phi_{ij}=\parallel log(P_{i}^{t})-\log(P_{j}^{t})\parallel_{2}.$$

*(Eq. S2)*

This metric allows the embedding to preserve global manifold structures while denoising local variations.

**LightGBM**

The Light Gradient Boosting Machine (LightGBM) classifier was trained to minimize the binary logistic loss. The model updates iteratively by adding a new decision tree $h_{m}\left( x \right)$to the existing ensemble $F_{m-1}(x)$:

$$F_{m}(x)=F_{m-1}(x)+\eta h_{m}(x)$$

*(Eq. S3)*

where $\eta$ is the learning rate scaling the contribution of the new weak learner.

Final predicted probabilities were obtained by passing the model output through a sigmoid function:


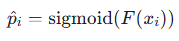


*(Eq. S4)*

These probabilities were subsequently projected onto the PHATE embedding to visualize the risk landscape.

**Structure of Nonlinear Additive Model (NAM)**

To ensure interpretability, the Nonlinear Additive Model (NAM) models each feature using an independent subnetwork. The final prediction is the additive sum of these subnetworks passed through a sigmoid activation:

$$\hat{y}=\sigma(\sum_{i} f_{i}(x_{i})+b)$$

*(Eq. S5)*

where $f_{i}(x_{i})$ represents the output of the subnetwork for the $i$-th feature, and $b$ is the bias term.

To evaluate the reliability of these probability predictions, we performed a calibration analysis by regressing the observed outcomes $y$ on the predicted probabilities $\hat{p}$:

$$y=\alpha+\beta\hat{p}+\varepsilon.$$

*(Eq. S6)*

This regression allows us to assess how well the predicted risk matches the actual observed risk.

**Loss Function for Variational Deep Embedding (VaDE)**

The model was trained by minimizing a composite loss function comprising a reconstruction term and a regularization term. Network Architecture & Training The encoder and decoder networks were constructed with two hidden layers containing 32 neurons each, utilizing ReLU activation functions. The model was trained for 100 epochs using the Adam optimizer with a learning rate of 10^-3^.

The total loss function, $\mathcal{L}$, is defined as follows:

$$\mathcal{L=}\mathcal{L}_{\text{recon}}+\beta\text{ }D_{KL}\left( q(z\mid x)\text{ }\parallel\text{ }p(z) \right)$$

*(Eq. S7)*

where $\mathcal{L}_{\text{recon}}$ denotes the reconstruction loss, calculated as the mean squared error (MSE) between the input 𝑥 and the reconstructed output x′.

$D_{KL}$ represents the Kullback–Leibler divergence between the approximate posterior distribution $q(z\mid x)$ and the prior distribution $\text{ }p(z)$.

The hyperparameter 𝛽 controls the strength of latent space regularization and was set to 0.1 in this study.

**Supplementary Table**

**Supplementary Table S1. Sensitivity analysis of PHATE-1 axis differences according to parameter variations**

| Diffusion Time (t) | Decay | Night Group Mean (SD) | Non-Night Group Mean (SD) | P-value |
| --- | --- | --- | --- | --- |
| 10 | 20 | 12.37 (31.06) | -11.87 (31.21) | < 0.001 |
| 10 | 40 | -17.83 (46.54) | 17.12 (44.20) | < 0.001 |
| 10 | 80 | -21.51 (57.23) | 20.65 (53.49) | < 0.001 |
| 30 | 20 | 3.51 (8.49) | -3.37 (8.77) | < 0.001 |
| 30 | 40 | 1.12 (2.68) | -1.08 (2.86) | < 0.001 |
| 30 | 80 | 7.32 (18.22) | -7.03 (18.04) | < 0.001 |
| 50 | 20 | 1.12 (2.68) | -1.08 (2.86) | < 0.001 |
| 50 | 40 | 2.64 (6.48) | -2.53 (6.48) | < 0.001 |
| 50 | 80 | 3.40 (8.37) | -3.27 (8.43) | < 0.001 |

Note: Regardless of the hyperparameter combination, the Night Shift group consistently showed a statistically significant difference in distribution along the PHATE-1 axis compared to the Non-Night Shift group.

**Supplementary Table S2. Performance comparison between the proposed NAM framework and conventional baseline models.**

| Model Type | Model Algorithm | Mean AUC (SD) | 95% CI | Key Characteristic |
| --- | --- | --- | --- | --- |
| Linear Model | Logistic Regression | 0.743 (0.018) | 0.708 – 0.778 | Low performance due to linearity assumption. |
| Black-box Model | Random Forest | 0.914 (0.012) | 0.890 – 0.938 | High accuracy but lacks interpretability (opaque). |
| Proposed Method | Neural Additive Model (NAM) | 0.864 (0.018) | 0.829 – 0.899 | Optimal balance between high non-linear accuracy and clinical interpretability. |

Note: While Random Forest achieved the highest AUC, it functions as a black-box model. NAM demonstrated significantly higher performance than Logistic Regression and provided interpretable shape functions essential for identifying clinical thresholds (e.g., TG > 2mmol/L), making it the most suitable choice for this study.

**Supplementary Table S3. Hyperparameters and Configuration Details for Analytical Models**

| Model | Parameter | Value / Setting | Description |
| --- | --- | --- | --- |
| PHATE | n_components | 2 | Embedding dimensions for visualization |
|  | knn | 5 | Number of nearest neighbors |
|  | decay | 40 | Alpha decay parameter (Selected via sensitivity analysis) |
|  | t (Diffusion time) | 30 | Optimal time scale (Selected from sweep: t=10, 30, 50) |
| LightGBM | objective | binary | Binary classification task |
|  | boosting_type | gbdt | Gradient Boosting Decision Tree |
|  | learning_rate | 0.05 | Step size shrinkage used to prevent overfitting |
|  | num_leaves | 31 | Maximum tree leaves for base learners |
|  | max_depth | -1 | Unlimited depth (controlled by num_leaves) |
| NAM  (Neural Additive Model) | Structure | [64, 64] | Two hidden layers with 64 units per feature subnet |
|  | Activation | ReLU | Rectified Linear Unit activation function |
|  | Optimizer | Adam | Adaptive Moment Estimation |
|  | Learning rate | 0.001 | Initial learning rate for optimizer |
|  | Loss Function | BCELoss | Binary Cross Entropy Loss |
| VaDE  (Variational Deep Embedding) | Architecture | VAE | Variational Autoencoder backbone |
|  | Input dim | 2 | Input dimensions (PHATE coordinates) |
|  | Hidden dim | 32 | Intermediate dense layer size |
|  | Latent dim | 5 | Dimension of the latent Gaussian space |
|  | n_clusters | 3 | Number of Gaussian components (Clusters) |
| General | Random Seed | 42 | Fixed seed for reproducibility |
|  | Validation | 5-fold | Repeated Stratified K-Fold (25 repetitions) |
